# Evolutionary trends of European bat lyssavirus type 2 including genetic characterization of Finnish strains of human and bat origin 24 years apart

**DOI:** 10.1007/s00705-015-2424-0

**Published:** 2015-04-17

**Authors:** Jakava-Viljanen Miia, Nokireki Tiina, Sironen Tarja, Vapalahti Olli, Sihvonen Liisa, Huovilainen Anita

**Affiliations:** Finnish Food Safety Authority Evira, Mustialankatu 3, 00790 Helsinki, Finland; Department of Virology, University of Helsinki, POB 21, Helsinki, Finland; Department of Veterinary Biosciences, Faculty of Veterinary Medicine, University of Helsinki, POB 66, FI-00014 Helsinki, Finland

## Abstract

Among other *Lyssaviruses,* Daubenton’s and pond-bat-related European bat lyssavirus type 2 (EBLV-2) can cause human rabies. To investigate the diversity and evolutionary trends of EBLV-2, complete genome sequences of two Finnish isolates were analysed. One originated from a human case in 1985, and the other originated from a bat in 2009. The overall nucleotide and deduced amino acid sequence identity of the two Finnish isolates were high, as well as the similarity to fully sequenced EBLV-2 strains originating from the UK and the Netherlands. In phylogenetic analysis, the EBLV-2 strains formed a monophyletic group that was separate from other bat-type lyssaviruses, with significant support. EBLV-2 shared the most recent common ancestry with Bokeloh bat lyssavirus (BBLV) and Khujan virus (KHUV). EBLV-2 showed limited diversity compared to RABV and appears to be well adapted to its host bat species. The slow tempo of viral evolution was evident in the estimations of divergence times for EBLV-2: the current diversity was estimated to have built up during the last 2000 years, and EBLV-2 diverged from KHUV about 8000 years ago. In a phylogenetic tree of partial N gene sequences, the Finnish EBLV-2 strains clustered with strains from Central Europe, supporting the hypothesis that EBLV-2 circulating in Finland might have a Central European origin. The Finnish EBLV-2 strains and a Swiss strain were estimated to have diverged from other EBLV-2 strains during the last 1000 years, and the two Finnish strains appear to have evolved from a common ancestor during the last 200 years.

## Introduction

Rabies is a fatal and incurable zoonotic disease caused by RNA viruses belonging to the genus *Lyssavirus* within the family *Rhabdoviridae*. The negative-sense lyssavirus genome encodes five proteins: nucleoprotein (N), phosphoprotein (P), matrix protein (M), glycoprotein (G) and RNA polymerase (L) in the order 3′-N-P-M-G-L-5′. Phylogenetic analysis and the virus-host relationship suggest that all 15 currently known lyssaviruses probably originated in bats and can be divided into phylogroups. Phylogroup I comprises the classical rabies virus (RABV) and the majority of bat lyssaviruses, whereas Lagos bat virus (LBV) and Shimoni bat virus (SHIBV) form phylogroup II [1, 2]. West Caucasian bat virus (WCBV), Ikoma lyssavirus (IKOV) and Lleida bat lyssavirus (LLEBV) may be representatives of a possible new phylogroups [3–5].

The first record of a rabid bat in Europe occurred in Germany in 1954 [6], but only with the advent of antigenic typing and molecular tools could European bat lyssavirus (EBLV) be characterised and distinguished from classical RABV and other lyssaviruses [7, 8]. After the death of a Swiss bat biologist in Finland in 1985 and subsequent characterisation of the causative agent, it became evident that bat rabies in Europe at the time was caused by two different lyssaviruses, EBLV-1 and 2 [7, 9]. Both of these viruses are genetically and antigenically related to RABV but are significantly different from each other. Early genomic sequencing also indicated that two distinct genetic lineages (a and b) of EBLVs have evolved, which appeared to cluster geographically [10, 11].

During 1977–2013, 1064 rabies cases were reported in 11 of the 45 known indigenous bat species in 16 European countries [12]. Although numerous human contacts with European bats, primarily from handling sick or injured animals, have been reported [13], only a few EBLV-induced human casualties have been conclusively demonstrated: in Voroshilovgrad, Ukraine (1977), in Belgorod, Russia (1985), in Finland (1985) and in the UK in Scotland (2002) [9, 14–17]. The Russian case was genetically typed as being EBLV-1a, while the Ukrainian case is only assumed to be EBLV-1 from antigenic profiling [15, 18]. The virus variants responsible for human rabies cases in the UK and Finland were typed as being EBLV-2a and b, respectively [17]. Subsequently, two other human rabies cases after a bat bite have been described in Ukraine [15]. However, these cases were diagnosed clinically and on the basis of anamnesis, and no virological or pathological results are available.

EBLV-2 is known to associate with two closely related *Myotis* bat species, Daubenton’s (*Myotis daubentonii*) and pond bats (*M. dascyneme*). The first isolation of EBLV-2 from a bat was carried out in 1987 from a pond bat in the Netherlands [19]. Elsewhere in Europe, EBLV-2 has been isolated sporadically from Daubenton’s bats in Switzerland [20], the UK [21, 22], Germany [23] and Finland [24]. In 1986, EBLV was detected in one Danish Daubenton’s bat and in one pond bat, but the causative strains were not analysed further. EBLV-2 was, however, demonstrated later in 2013 in mouth swabs from two Danish Daubenton’s bats using a molecular diagnostic strategy [25]. The strains from the UK and from the Netherlands appear to be closely related, as are the strains from Germany and those isolated from Switzerland [26]. The Finnish EBLV-2 strains and a strain from Switzerland have been suggested to form a lineage designated as EBLV-2b, with rest of the isolates forming the lineage EBLV-2a [10, 11]. EBLV-2 strains appear to form clusters according to the geographical area and year of isolation.

Evolutionary studies on lyssaviruses have tended to focus on the N protein, a conserved structural protein, and the G protein, which contains domains responsible for host-cell receptor recognition and membrane fusion, and which is the major target for the host neutralizing antibody response [1]. Previously, comprehensive evolutionary analysis has demonstrated that EBLV-1 and EBLV-2 have differing population structures and dispersal patterns. Molecular-clock estimates have suggested that the current linage of EBLV-1 arose some 500 to 750 years ago [27]. Little is known about the forces shaping the evolution of EBLV-2, and only three strains of EBLV-2 have previously been fully sequenced (EU293114, EF157977 and KF155004).

Finland has been free of rabies in terrestrial mammals since 1991. Nevertheless, rabies can remain a residual risk to public health due to the natural circulation of EBLV-2 [24, 28]. Here, we describe the phylogenetic analysis of two Finnish EBLV-2 strains, the first isolated from a human in 1985 and the second from a diseased bat in 2009. Using these isolates as a calibrator, this study provides an estimate of the EBLV-2 molecular clock. Increased information on the genome sequences of lyssaviruses is fundamental to understanding their epidemiology and evolution, and to demonstrating the importance of continuous surveillance and molecular characterization of lyssaviruses circulating in animal populations.

## Materials and methods

### Virus isolation in MNA cells and in suckling mice

#### Ethical approval and permission

The National Animal Experiment Board of the County Administrative Board of Southern Finland approved the diagnostic mouse inoculation test (permission number ESLH-2008-06899/Ym-23), which followed Finnish legislation, namely, the Finnish Act on the Use of Animals for Experimental Purposes (62/2006).

Human bat virus isolate FI-85 [9, 16] used in this study was the first archived newborn-mouse passage and had been kept at -70 °C since 1986. Virus isolation of the bat virus isolate FI-09 [24] was attempted in MNA cell culture (mouse neuroblast cells, Neuro-2a [ATCC^®^ CCL-131™]), with three additional passages according to a procedure described in the OIE manual [29]. The bat brain suspension was subsequently inoculated into suckling mice. Eight newborn mice (ScaNmri) at 2 days old were intracerebrally infected with 20 µl of the suspension in a BSL-3 laboratory. When the mice started to develop signs of encephalitis, they were euthanized and the brains were collected and examined using a fluorescence antibody test (FAT) for the presence of lyssavirus as described earlier by Jakava-Viljanen et al. [24].

### RNA extraction, primer design and RT-PCR

RNA was extracted from the brain suspensions of the mice using a QIAamp Viral RNA Mini Kit (QIAGEN, Germany, Hilden) according to the manufacturer’s instructions. Primers (Table 1) were designed with the program PCR Suite [30] and modified afterwards, if needed. A OneStep RT-PCR Kit (QIAGEN, Hilden, Germany) was used to amplify 34 overlapping fragments. The following thermal profile was used: a single cycle of reverse transcription for 30 min at 50 °C, 15 min at 94 °C for reverse transcriptase inactivation and DNA polymerase activation, followed by 30 amplification cycles of 1 min at 94 °C, 1 min at 50 °C and 1 min at 72 °C. After agarose gel electrophoresis, the bands were cut from the gel and DNA was extracted using a QIAquick Gel Extraction Kit (QIAGEN, Hilden, Germany).

**Table 1:** Primers used in the RT-PCR and sequencing. The position numbering is according to the EBLV-2 isolate RV1333 (EF157977)

Primer	Sequence (5’-3’)	Position
1f	acg ctt aac gac aaa acc ag	1 - 20
1r	tag ctc tcc caa tcg tca gg	326 - 345
2f	cgc tag gtt gga tcc tga tg	256 - 275
2r	ggc gca cat ctt gtg agt ag	636 - 655
3f	cca acg tag ctg aca gaa tgg	558 - 578
3r	aca tct cgt gag gtg cac ag	1066 - 1085
4f	cgg gag tta cat ggg tca ag	1015 - 1034
4r	gtc tgg cct gat gat tcg ag	1354 - 1373
5f	cag gat cat ggt caa tgg	1291 - 1308
5r	tcc caa cac cct caa ggt ag	1812 -1831
6f	aag aag aag gaa gcg atg agg	1745 - 1765
6r	tgc gct att tct gct tca ac	2116 - 2135
7f	acc tgc gct gga atg gtc	2070 - 2087
7r	ggg agc cat agg tca tca tc	2591 - 2610
8f	agt gag agg ttg cag gga tg	2530 - 2549
8r	act ctg ccc att gaa aca cc	2869 - 2888
9f	ttc cag agg gaa tga act gg	2826 - 2845
9r	ggt gtt cag tcg ggt gtt tc	3245 - 3264
10f	ctt tta tga gca ata gaa caa aac c	3186 - 3210
10r	atg tcg gat cac ctg cag tc	3688 - 3704
11f	aac tac cac gtt caa gag aaa gc	3619 - 3641
11r	ttt gcc tca tcg tga ttt tg	4115 - 4134
12f	tga aac tgt gtg gaa tct ctg g	4059 - 4080
12r	atg ctg ttg aag cat tgc ag	4518 - 4537
13f	tcc tca tca caa tgg agt ctt c	4441 - 4462
13r	ccc act ttg gga agt gac ag	4830 - 4849
14f	aaa gag agc caa acc caa cc	4787 - 4805
14r	att gca tcc tct ccc act tg	5154 - 5171
15f	acc ggt aca cag ggt ctt gc	5076 - 5095
15r	gca tct atc tcc ggt tcg ac	5458 - 5477
16f	aga tga ttg atc ccc tgg ag	5414 - 5433
16r	gag gca ctt tcg act tct gg	5747 - 5766
17f	cgc aca atc cat gat gtc tc	5697 - 5716
17r	gaa tca gga ggg agt tga acc	6173 - 6193
18f	tct cag agt gcc aac tgt ctg	6106 - 6126
18r	gtt cct tca agc tgg ctc ac	6415 - 6434
19f	tta gtg cag agg gct gaa gg	6340 - 6359
19r	tat ggg atc aaa ggg tgg tc	6709 - 6728
20f	ctg gct aaa cgg atc ctc ag	6634 - 6653
20r	aag aat tcc ctg ggg ttg ac	6964 - 6983
21f	ccg tcc cca gtg aga aag tc	6917 - 6936
21r	gac ctt gtc ccg tga ctc tg	7209 - 7228
22f	ttg gcg aac tac atc tta ccc	7129 - 7149
22r	tga gtc cct ctt ggg tca ac	7641 - 7660
23f	agc aca ggg aga caa cca ag	7590 - 7609
23r	gtg aaa tac cgc ctg gac tg	7979 - 7998
24f	gtc gca cag cat tca caa tc	7924 - 7943
24r	agc aga atg gtt gga ctt gc	8332 - 8351
25f	ccg gac ttg ggt gat aga ag	8254 - 8273
25r	aaa ttg ccg tcg aat tgt tc	8567 - 8586
26f	gct cat cct tcc tcg gaa tac	8513 - 8533
26r	gat ttg agt ccc tgg caa tg	9021 - 9040
27f	cca acg tcc atg ttg tca ag	8966 - 8985
27r	aga cat ccg gga aca tga ag	9417 - 9436
28f	caa gtg cat ccg acc gat ag	9369 - 9388
28r	cag atc gaa gtg agg gtt cc	9831 - 9850
29f	tgt tga ggc tag aca atc atc c	9788 - 9809
29r	taa ggt gtc ttc ccc gtg ac	10151 - 10167
30f	atc cga ctc agg cag ttg ag	10105 - 10124
30r	gag gcc atg agg tca ttc ac	10606 - 10625
31f	tgg aat ctc cag aac tgt gc	10539 - 10558
31r	tgg cct tgt agt ctg ggt tc	10923 - 10942
32f	ctc tcg atc aat ggt cca ctc	10867 - 10887
32r	tta gcc aag gtc cct ctt tg	11287 - 11306
33f	tga agt cga gtc att cct agt cc	11238 - 11260
33r	gct act acc ggc aag tcg ag	11672 - 11691
34f	aag caa gtc ata cga gga ag	11533 - 11552
34r	acg ctt aac aaa aaa aac ata g	11909 - 11930

### Sequencing and sequence analysis

PCR products were sequenced using an ABI 3100 Avant Genetic Analyser (Applied Biosystems) with the primers used in the PCR and a Big Dye Terminator v3.1 Cycle Sequencing Kit (Applied Biosystems). Before sequencing, the reaction products were purified using a DyeEx 2.0 Spin Kit (QIAGEN, Hilden Germany). The sequences were analysed with Sequencing Analysis software version 6.0 (Applied Biosystems).

### Phylogenetic analysis

The newly determined sequences (GenBank accession numbers JX129232 and JX129233) were aligned with other published full-length lyssavirus sequences from bat-type isolates obtained from GenBank to evaluate the genetic diversity of EBLV-2 strains and their relationship to other bat-associated genotypes. Amino acid sequences were deduced using the “translate” function of the program MEGA v.5.1 [31]. Phylogenetic trees of full-length genomes and partial N coding sequences were calculated using the maximum-likelihood approach in the program MEGA with 1000 bootstrap replicates. The pairwise sequence identities were calculated using CLUSTAL W2 (http://www.ebi.ac.uk/Tools/msa/clustalw2/), with default settings.

### Rate of evolution and time of divergence

The program BEAST [32] was used to estimate the substitution rate of EBLV-2 and time of divergence from the phylogenetically closest lyssaviruses. The data set used for estimating the rate of evolution was based on the partial N coding sequences (400 bp) of EBLV-2 strains isolated from the years 1986 to 2012. The HKY model of nucleotide substitution was used [33] with both strict and relaxed lognormal clock models. Convergence of parameters was assessed using TRACER [32], and each run was continued until the effective sampling size of all parameters was greater than 200.

## Results

### Isolation of the EBLV-2 FI-09 strain in MNA cells and suckling mice

Inoculation of the bat sample into MNA cells revealed the presence of virus in only a limited number of cells after 3 days of incubation, and after the three subsequent passages, the tissue culture infection test was negative. When the cell culture isolation was unsuccessful, the brain suspension of the bat was inoculated into suckling mice. The mice began to develop signs of encephalitis on day 12 p.i. By day 17 p.i., all eight mice were euthanized. The presence of the virus in all of the brain samples was confirmed by FAT.

### EBLV-2 genome, genomic distance and phylogenomic pattern

The complete lengths of the FI-85 and FI-09 genomes were 11,928 and 11,927 nucleotides, respectively (GenBank accession numbers JX129233-JX129232). Twenty nucleotides at the 5’ end and 22 nucleotides at the 3’ end of the genomes were primer-derived and were excluded from all phylogenetic analyses.

The general genome organization was typical for lyssaviruses, consisting of five structural genes, N, P, M, G and L, and non-coding regions between them and at both ends of the genome. The lengths of the genes and the number of deduced amino acids (in parentheses) were 1356 (451 aa), 894 (297 aa), 609 (202 aa), 1575 (524 aa) and 6384 (2127 aa), respectively. The long untranslated region between the G and L genes was the only area that varied in length between the two Finnish EBLV-2 strains, being 510 bp in FI-85 and 509 bp in FI-09. The corresponding lengths for other completely sequenced EBLV-2 strains were 511 bp for EU293114 and 512 bp for KF155004 and EF157977.

**Fig. 1:**
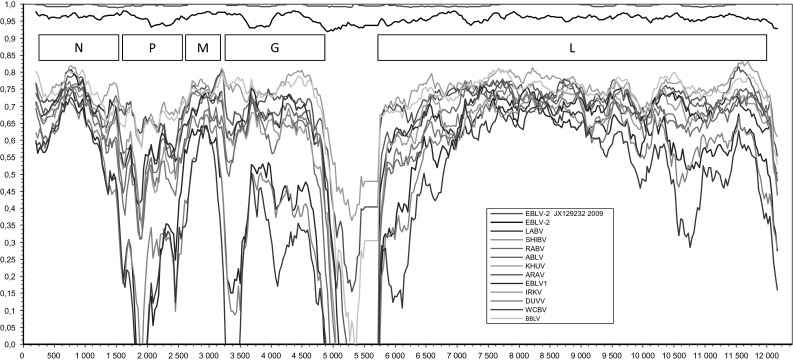
Similarity plot of bat-related lyssavirus genomes. Similarity is shown on the y-axis, and the position in the genome is shown on the x-axis

The nucleotide sequence identity between the FI-85 and FI-09 strains was 99.6 %. There were four amino acid differences between the two Finnish strains: N gene aa 106 (N><S), G gene aa 158 (V><A), L gene aa 154 (R><K) and aa 1656 (G><D). The N, P, M, G and L genes of the FI-85 and FI-09 strains shared 98–99 %, 98 %, 99–100 %, 97–99 % and 99 % amino acid sequence identity, respectively, with the previously published EBLV-2 strains. The similarity plot in Figure 1 displays the similarities along the genome. The non-coding regions are highly divergent, yet rather similar for all EBLV-2 strains.

### Phylogenetic analysis and evolutionary trends

**Fig. 2:**
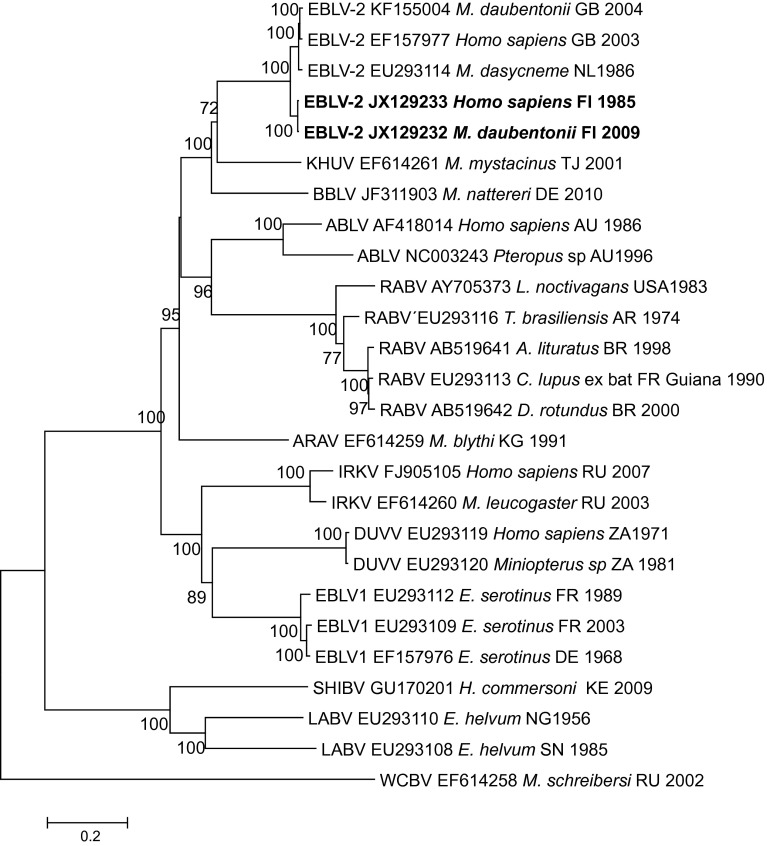
Phylogenetic tree of lyssaviruses estimated using complete coding sequences. The phylogenetic tree was calculated using the maximum-likelihood approach in the program MEGA [30] with 1000 bootstrap replicates

A phylogenetic tree was constructed based on the genome sequences of the two new EBLV-2 isolates and the full-length genome sequences of bat-related lyssavirus obtained from GenBank (Fig. 2). The analysis revealed that all five EBLV-2 strains are monophyletic. The phylogenetic tree also demonstrated that EBLV-2 strains share the most recent common ancestry with BBLV and KHUV, and more distant ancestry with ARAV, RABV and ABLV (albeit with low bootstrap support). A second major cluster of lyssaviruses consists of IRKV, DUVV and EBLV-1. The clearly most divergent group is formed by SHIV, LBV and WCBV.

**Fig. 3:**
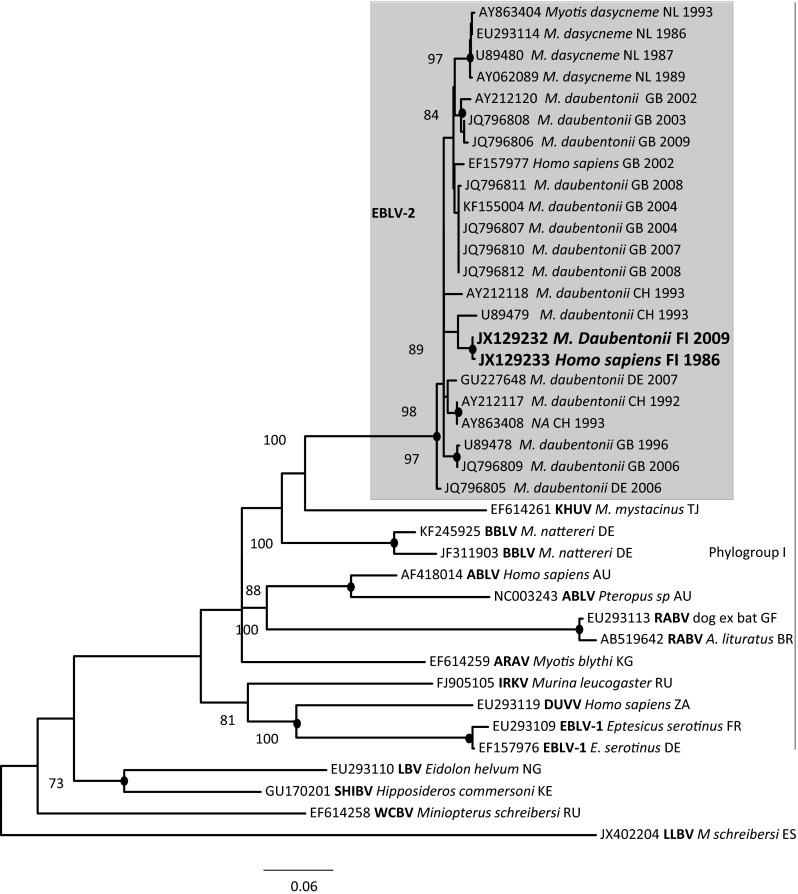
Phylogenetic tree based on partial N gene (400 nt) sequences. The phylogenetic tree was calculated using the maximum-likelihood approach in the program MEGA [31] with 1000 bootstrap replicates

In order to include more EBLV-2 strains, we constructed a second phylogenetic tree (Fig. 3) based on partial N gene sequences (400 nt), which are much more abundant in the GenBank database. EBLV-2 strains were also monophyletic in this region. Some discrepancies were observed between the two trees, especially in the composition of the major clusters, which were not reproduced in the phylogeny based on partial N gene sequences.

**Fig. 4:**
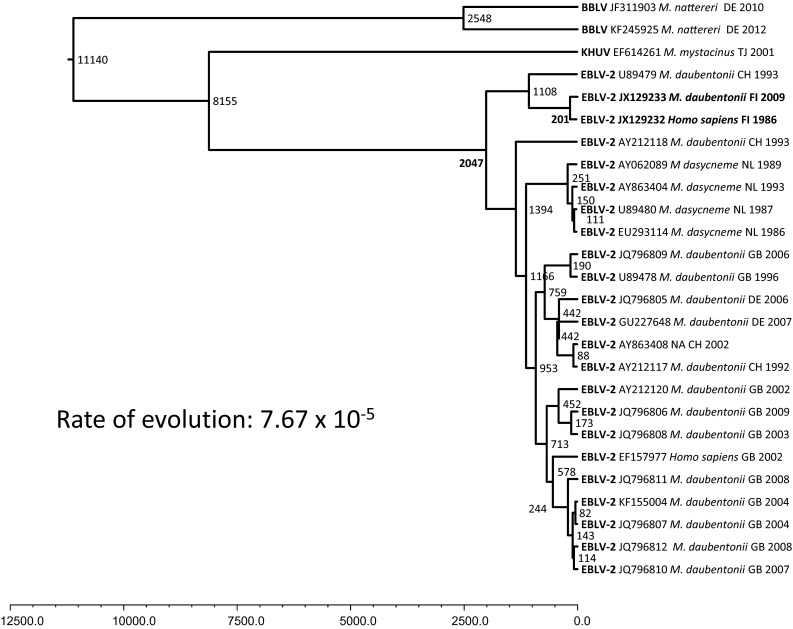
Phylogenetic tree of EBLV-2 based on partial N gene (400 nt) sequences with a molecular-clock estimate. The program BEAST [32] was used to estimate the substitution rate of EBLV-2 and time of divergence from the phylogenetically closest lyssavirus. The HKY model of nucleotide substitution was used [33] with both strict and relaxed lognormal clock models. Convergence of parameters was assessed using TRACER [31], and each run was continued until the effective sampling size of all parameters was greater than 200

The substitution rate of European bat lyssavirus-2 was estimated using the two Finnish strains isolated 24 years apart as a calibrator. The overall rate was 7.67 × 10^−5^ substitutions per site per year. The current diversity of EBLV-2 was estimated to have appeared during the last 2000 years. However, EBLV-2 and other phylogroup I viruses were estimated to have already diverged from other lyssaviruses at about 8000 years ago (Fig. 4). The divergence of Finnish EBLV-2 strains and a strain from Switzerland from other EBLV-2 strains has occurred during the last 1000 years. The two Finnish strains have evolved from a common ancestor during the last 200 years.

## Discussion

Sequencing, phylogenetic analysis and evolutionary analysis of Finnish EBLV-2 strains isolated from a human case in 1985 and from a bat in 2009 were described. It was shown that the EBLV-2 isolates share high nucleotide and amino acid sequence identity, regardless of the year and origin of isolation, but they also cluster according to the host species and the geographical place of isolation.

The closest relatives to EBLV-2 (Bokeloh and KHUV) are lyssavirus strains from *Myotis* bats from Germany and Tajikistan. An interesting case is ARAV (originating from Kyrgystan), which has an uncertain position in the phylogenetic analysis. The possibility of recombination of rabies viruses has been demonstrated in earlier studies [34–36]. Lleida bat lyssavirus, detected in a bent-winged bat (*M. schreibersii*) in Spain in 2011 [5], is the most unique lyssavirus in Europe based on the N gene, although the whole genome sequence is not available. WCBLV has also been detected in *M. schreibersii*, and antibodies against WCBV have been detected in Africa in *Miniopterus* bats [37]. The tree topology was somewhat different in the phylogeny based on either the complete coding sequences or the commonly used partial N sequences, and it should be appreciated that for a detailed analysis of evolutionary history, complete genomes should be used. The limitation of this study, as in other studies, is the amount of data available for comparison.

The nucleotide sequence identity (deduced amino acid sequence identity) between FI-85 and FI-09 was 99.6 % (99.8 %). The N, M and L proteins were similar in structure and length between members of different lyssavirus species and strains, whereas the lengths of the P and cytoplasmic regions of the G protein were variable [38–40]. No differences in structure were seen in the Finnish EBLV-2 isolates in comparison to other EBLV-2 complete genomes [40–42]. The similarity plot in Figure 1 further demonstrates the similarities along the genomes of different lyssaviruses. Interestingly, the non-coding regions, which are highly divergent in other lyssaviruses, were rather similar for all EBLV-2 strains.

Based on this study, we estimate that the Finnish EBLV-2 strains and one Swiss strain from among the other EBLV-2 strains have evolved from a common ancestor during the last 1000 years. Earlier, the Finnish strains and the strain from Switzerland were suggested to form the lineage EBLV-2b, whereas the rest of the isolates form the lineage EBLV-2a [10, 11, 20, 27]. In our study, there was a division into subgroups EBLV-2a and EBLV-2b when full genomes were compared (Fig. 2), but when more strains were compared using shorter, 400-bp N gene sequences, we could not confirm the suggested division into subgroups (Fig. 3). In our comparison, the Finnish strains and the strain from Switzerland also clustered together based on N gene analysis. Interpretation of the molecular epidemiology of the strains is further complicated by the history of the Swiss bat biologist who died in Finland in 1985 of EBLV-2 infection (JX129233), who had been bitten 51 days before the onset of clinical signs by a Daubenton’s bat that was abnormal. The bat was freed before the patient developed symptoms and was not available for rabies analysis [16]. However, it is possible that the bat originated from Central Europe, possibly from Switzerland, even though the exposure took place in Finland.

The molecular clock estimates suggest that the current linage of EBLV-2 arose some 2000 years ago and that the most recent common ancestor (MRCA) occurred around 8000 years ago. In earlier molecular clock studies, Tao et al. [43] estimated based on G gene analysis that the time to the most common ancestor (TMRCA) of all lyssaviruses was approximately 5030 years. The West Caucasian bat virus divided first, and then phylogroup I and phylogroup II divided about 4000 years ago [43]. Davis et al. [27] estimated that the MRCA of EBLV-1 existed around 500 to 750 years ago based on G and N sequences, or 70 to 300 years ago, depending on the substitution rate [44]. The molecular clock estimates cannot be used to precisely describe events that occurred thousands, or even tens of thousands of years ago, since sequence data only exist from the last few decades. The overall rate of evolution appears somewhat slower for the bat-type lyssaviruses than for RABV. This is in line with bats being considered the true reservoir of lyssaviruses. The overall evolution rate for EBLV-2 in our study was 7.67 × 10^−5^ substitutions per site per year. For RABV, it has been suggested to be 1.56–1.78 × 10^−4^ [45], and 1 × 10^−3^ to 5.5 × 10^−4^ [7, 43, 46, 47], and for EBLV-1, 5–6 × 10^−5^ to 5–6 × 10^−4^ [27].

Behavioural and ecological traits of bats, such as hibernation, migration and coloniality, might influence viral evolution. Some rabies virus lineages evolve up to 22 times faster than others, depending on the reservoir host [48, 49]. Virus lineages in bat species in the temperate climate zone were found to have four times slower rates of evolution than lineages in bats from tropical or subtropical climates, and viral evolution was faster in bats that remain active year-round in comparison to bats that hibernate. This could be a major factor in a cold climate region such in Finland. Viral evolutionary rates were similarly unconstrained by host evolutionary relatedness such that lyssaviruses associated with closely related bat species or subspecies often had dissimilar evolutionary rates. Streicker et al. [48] demonstrated that the local host environment determines the evolutionary rates of lyssaviruses. Daubenton’s bats are facultatively seasonal migrants covering middle-range distances of 100–150 km [50], which supports the clustering of EBLV-2 according to the geographical place of isolation. In Finland, antibodies to EBLVs have only been detected in a restricted geographical area close to the location where the EBLV-2-positive bat was found in 2009 [28]. In a study of Daubenton’s bats from Finland, the UK, Switzerland and Spain, no population structuring was observed [51]. Daubenton’s bats migrate between the UK and the mainland, and the genetic structure of Daubenton’s bats is relatively homogeneous in western parts of Europe [52]. This could explain the relatedness within the proposed lineage 2a. As the migration pattern of Daubenton’s bats does not support the finding that the Finnish EBLV-2 Daubenton’s bat strain is closely related to the EBLV-2 strain from Switzerland, one can speculate whether there could have been contact between indigenous Finnish bats and the bats that were freed in Finland and whose origin is unknown [9, 16].

Increased information on the complete genome sequences of lyssaviruses is fundamental to understanding their epidemiology and evolution and demonstrates the importance of continuous monitoring and molecular characterization of lyssaviruses circulating in human and animal populations.
